# Future changes in the risk of compound hot and dry events over China estimated with two large ensembles

**DOI:** 10.1371/journal.pone.0264980

**Published:** 2022-03-24

**Authors:** Zhenfei Tang, Ting Yang, Xin Lin, Xinxin Li, Rong Cao, Wei Li

**Affiliations:** 1 Joint International Research Laboratory of Climate and Environment Change, Collaborative Innovation Center on Forecast and Evaluation of Meteorological Disaster, Nanjing University of Information Science and Technology, Nanjing, China; 2 Fujian Climate Center, Fujian Province Meteorology Bureau, Fuzhou, China; 3 Fujian Key Laboratory of Severe Weather, Fujian Province Meteorology Bureau, Fuzhou, China; 4 Fujian Meteorological Service Center, Fujian Province Meteorology Bureau, Fuzhou, China; Texas A&M University, UNITED STATES

## Abstract

Under the context of global warming, compound dry and hot events (CDHEs) will increase and bring serious losses to society and the economy. The projection of CDHEs is of great significance for policy-making and risk assessment. In this paper, two large ensemble simulations, CanESM2-LE and CESM-LE, are used to estimate the risk of extreme CDHEs under different warming scenarios in China. First, the biases of the model in the simulation of the temperature and precipitation over the China region are corrected, and the index of CDHEs is established based on a copula function. The results show that extreme CDHEs will occur more often in China with the increase in global warming and the more severe extreme CDHEs are, the greater the risk will be in the future with higher uncertainties. Events that would be attained once every 50 and 100 years in the current climate from CESM-LE (CAanESM2-LE) will be 1.2/1.6 (1.1/1.5) times and 1.3/2.3 (1.5/2.0) times more likely to occur in a 1.5°C/2.0°C warmer climate, respectively. Northwestern China will experience the greatest increase in the risk of extreme CDHEs. Extreme CDHEs expected once every 100 years in the current period over NW China are expected to occur approximately every 5 and 4 years under a 4.0°C warmer world in CanESM2-LE and CESM-LE, respectively.

## 1. Introduction

Global warming caused by greenhouse gas emissions from human activities is the main feature of climate change in the last century. In the context of warming, many regions in different areas of the world have experience more and more extreme weather events. The main features include increases of extreme high temperatures and decreases of extreme low temperatures. Extreme precipitation and drought will also increase in the future [1]. Frequent extreme weather events will have a significant impact on human life and the social economy. Between 1999 and 2018, more than 12,000 extreme weather events directly caused approximately 495,000 deaths worldwide and losses of US$ 3.54 trillion [2]. However, due to the potential feedback effect of physical processes within the climate system, different types of extreme climate events have combined effects, thus increasing the climate impact to some degree [3, 4]. As a result, traditional measures of the impact of a single extreme event may underestimate the impact of climate change due to the influence of compound events.

Extreme dry and hot events are the two most important climate disasters on a global scale because of their profound impact on crops and people’s lives [5]. Based on observations, both types of extreme events are increasing in most regions of the world [6–8]. Increasing temperature results in an increase in evaporation, which intensifies the severity of drought [9]. As the surface dries, it heats the atmosphere by increasing the transfer of sensible heat from the Earth’s surface. Observations and model results show that the positive feedback effect of temperature and precipitation is very obvious in summer at middle and high latitudes [10]. Therefore, the strong coupling relationship between temperature and precipitation may increase the frequency of compound dry and hot events (CDHEs) [11, 12]. For example, CDHEs caused serious economic and property losses and human casualties in Europe in 2003, Russia in 2010 and California in 2014 [13–15].

In the Chinese region, extreme temperature and drought have been well studied as univariate events, including characteristics of changes under the current climate and projected changes under future emission scenarios [16, 17]. For example, the frequency and intensity of extreme heat has increased significantly over much of China. Drought is increasing in northern China and decreasing in the southern China [18]. As the world continues to warm in the future, the intensity and frequency of extreme heat and drought will further increase. In recent years, many studies have found that the frequency and intensity of CDHEs in China are also increasing [16, 19]. However, researches on future changes in CDHEs, especially for extreme events (such as those that occur once in 50 or 100 years), are still limited. The projection of the risk of extreme CDHEs is of great significance for disaster prevention and mitigation.

There are usually two methods to estimate the probability of extreme events [20]. One is based on the generalized extreme value distribution model, which extrapolates extreme events that have not occurred historically, but the extrapolation method requires knowledge of the distribution function in advance. The second method is to directly use large sets of sample data and the method of empirical estimation [21]. Recently, Large ‘‘initial-condition” ensemble experiments have been developed, which provides a satisfactory sampling of extreme event, which can allow us to estimate the probability of extreme event without any probability distribution. In this paper, two sets of large sample models, CanESM2-LE and CESM-LE, are used to estimate the risk projections under different warming scenarios. As the possibility of limiting global warming to 2°C is becoming increasingly low, it is necessary to analyze the risk projections under higher warming to provide a scientific basis for climate change policy formulation and risk assessment.

## 2. Data and methods

### 2.1. Data

Two large sets of sample data are used in this paper: the Canadian Earth System Model Large Ensemble (CanESM2-LE) and the Earth System Model large ensemble (CESM-LE). The spatial resolution of CanESM2-LE is 2.8°×2.8°, and 50 members simulated the Earth’s atmosphere under the same radiative forcing but different initial conditions during 1950–2100 [22, 23]. The different initial conditions were derived from five different simulations of the historical period 1850–1950. For each simulation, 10 ocean-atmosphere coupling simulations were performed in 1950 with random perturbation initial conditions. A total of 5×10 simulated atmospheric conditions over 150 years were obtained. The historical forcing was used from 1950 to 2005, and the RCP8.5 forcing was used after 2006.

The second large initial-condition ensemble dataset was CESM-LE. CESM-LE consists of 40-member ensembles of fully coupled Community Earth System Model version 1 (CESM1) simulations for the period 1920–2100 [24]. All simulations are performed at a horizontal resolution of approximately 1 degree in latitude and longitude. Each member is subject to the same radiative forcing (historical forcing up to 2005 and RCP8.5 forcing to 2100) but begins from a slightly perturbed initial temperature field within the range of round-off errors for each realization. More description of the CESM is given in Kay et al. [24].

**Fig 1:**
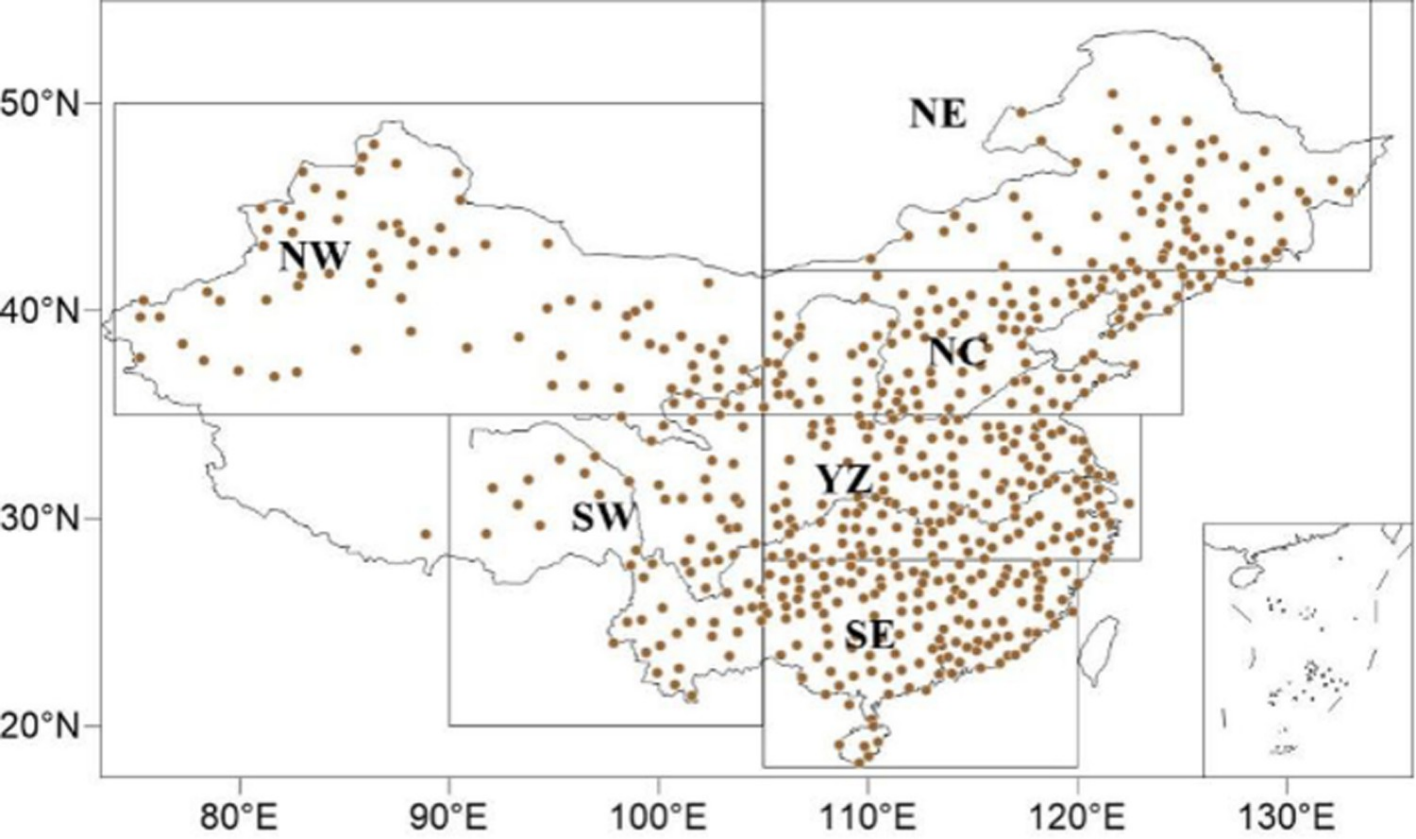
Locations of 603 stations used in this study and the six subregions of China: Northwest China (NW: 35° - 50°N, 74° - 105°E), Southwest China (SW: 20° - 35°N, 90° - 105°E), Northeast China (NE: 42° - 55°N, 105° - 134°E), North China (NC:35° - 42°N, 105° - 125°E), the Yangtze River Valley (YZ: 28° - 35°N, 105° - 123°E), and Southeast China (SE:18° - 28°N, 105° - 120°E).

Due to the coarse resolution of climate models and their inability to reproduce the key physical processes that influence extreme events, there are large biases in the simulation of extreme events on a regional scale. Therefore, before using a model to carry out future risk projection, the bias correction method is used to correct the simulation biases based on observational data. The observational data used in this paper are from the daily temperature and precipitation records of 603 meteorological stations in China from 1961 to 2015 that are provided by the National Climate Center of China Meteorological Administration [25]. China is a vast country with complex topographic and climatic conditions. To better study the changes in CDHEs in China under different warming conditions in the future, this paper divides China into 6 subregions according to the distribution of stations and China’s physical geography [26], as shown in Fig 1: Northwest China (NW: 35° - 50°N, 74° - 105°E), Southwest China (SW: 20° - 35°N, 90° - 105°E), Northeast China (NE: 42° - 55°N, 105° - 134°E), the Yangtze River Valley (YZ: 28° - 35°N, 105° - 123°E), and Southeast China (SE: 18° - 28°N, 105° - 120°E).

### 2.2. Methods

#### 2.2.1. Definition of compound dry and hot even

In this paper, a copula model is used to define the compound dry and hot event at monthly scale [27]. The copula model (C) of two random variables, the JJA precipitation A and the JJA temperature B, can be expressed as

P(A≤a,B≤b)=C[F(a),G(b)] (1)

where F(a) and G(b) are marginal functions of the two variables, respectively.

Many copula models are used to simulate the dependent structure between two random variables, including Gaussian, Frank, Gumbel, and Clayton. In this paper, the Gaussian model is used to establish relations between hot and dry events.

We first estimate the marginal distribution of mean summer temperature and precipitation using the empirical estimation method. Dry events are defined as precipitation less than or equal to a threshold, and hot events are defined as temperatures greater than or equal to a threshold. Thus, the probability of dry and hot events can be obtained:

p=P(A≤a,B≥b)=F(a)−C[F(a),G(b)] (2)

The frequency of CDHEs can be used as a measure of the severity of compound dry and hot events. To compare the severity of compound dry and hot events over different periods, the occurrence frequency is normalized to the index SDHI, which represents the severity of the composite events by fitting the normal distribution F:

$${SDHI=\emptyset }^{-1}[F(p)]$$ (3)

where ϕ is the standard normal distribution and F is the marginal cumulative distribution, which remaps the joint probability to the uniform distribution. The smaller the SDHI value is, the stronger the compound dry and hot event intensity is.

The contribution of temperature and precipitation to the changes in the SDHI was investigated using multiple regression.


SDHI=a×P+b×T+C (4)

The future changes of SDHI can be calculated by:

ΔSDHI=aΔP+bΔT (5)

Where, ΔP and ΔT are the change in the mean precipitation and temperature. Then the relative contribution (%) of precipitation and temperature to PI can be got by:

$$P_{c}=\frac{a\Delta P}{\Delta SDHI}\times 100$$ (6)

$$T_{c}=\frac{b\Delta T}{\Delta SDHI}\times 100$$ (7)

$$P_{c}+T_{c}=100\%$$ (8)

The percentage of the relative contribution by *P*_*rc*_ and *T*_*rc*_ can be calculated as:

$$P_{rc}=\frac{\left|P_{c}\right|}{\left|P_{c}|+|T_{c}\right|}\times 100$$ (9)

$$T_{rc}=\frac{\left|T_{c}\right|}{\left|P_{c}|+|T_{c}\right|}\times 100$$ (10)

#### 2.2.2. Determining the temperature increase threshold

To explore the risk of extreme drought from different levels of warming, scenarios with 1.5°C, 2°C, 3°C and 4°C warming above preindustrial levels were considered. CESM-LE calculated the preindustrial global mean surface temperature (GMST) in its first run, and we calculated GMST of CanESM2-LE for the preindustrial period 1860–1900. The moving window was set as 21, and the time series of GMST relative to preindustrial anomalies were processed by moving average filtering to find the first year that reached the determined warming level. A total of 21 years before and after the first year were taken as the climate of this warming level. We used 1°C as the current climate because the current climate warming was 1°C more than the industrial period (World Meteorological Organization, 2019). The period when the global mean surface temperature reaches 1°C, 1.5°C, 2°C, 3°C and 4°C compared to the preindustrial period for CanESM2-LE (CESM-LE) are 1990–2010 (2006–2026), 2004–2024 (2020–2040), 2017–2037 (2032–2052), 2038–2058 (2051–2071) and 2056–2076 (2070–2090), respectively.

#### 2.2.3. Bias correction

Bias correction method is an important technology in reducing the simulation biases [28, 29]. In this paper, the quantile mapping (QM) method is used to correct model biases [30].

The relation of CDF between the observation (*P*_*o*_) and simulation (*P*_*m*_) was firstly constructed:

$$F_{o}(P_{o})=F_{m}(P_{m})$$ (11)

A transfer function of CDF between the model and observed precipitation can be obtained:

$$P_{O}=F_{O}^{-1}(F_{m}(P_{m}))$$ (12)

This transfer function will remain valid in the future, and the bias corrected simulation can be calculated in the future period.

The daily precipitation and temperature data observed during 1985–2005 were used as model calibration standards to calibrate the model data with similar warming levels. The years 1992–2012 of CESM-LE and 1978–2008 of CanESM2-LE were selected as the periods with similar warming levels. The local intensity calibration (LOCI) was used to make the number and intensity of rain days simulated by the model consistent with the observations. We interpolated the model data to the site and then corrected the biases of each station in each member of the model during the current climate (1998–2018) and future climate data of the model.

#### 2.2.4. Probability and risk ratio

In this paper, an unbiased estimation formula is used to calculate the occurrence probability and risk ratio of high temperature and drought events under different climate conditions and warming levels [31]. The probability of occurrence is calculated as follows:

$$p=\frac{m-0.4}{N+0.2}$$ (13)

where N represents the total number of years, and m is the rank of the SDHI value.

The risk ratios for events under different climatic conditions are calculated, and the changes in the risk of extremely high temperatures are described, as defined below:

$$PR=\frac{P_{1}}{P_{0}}$$ (14)

where *P*_0_ is the probability of CDHEs under current climate conditions and *P*_1_ is the probability of CDHEs under different warming climate. *PR*>1 represents an increasing risk of the occurrence of extreme CDHEs.

The confidence interval of PR was calculated based on the resampling-based bootstrap technology. The uncertainty range between 5% (2.5%) and 95% (97.5%) quantile of 1000 bootstrap was used to demonstrate 90% (95%) confidence level.

## 3. Result

### 3.1. Mode performance evaluation

**Fig 2:**
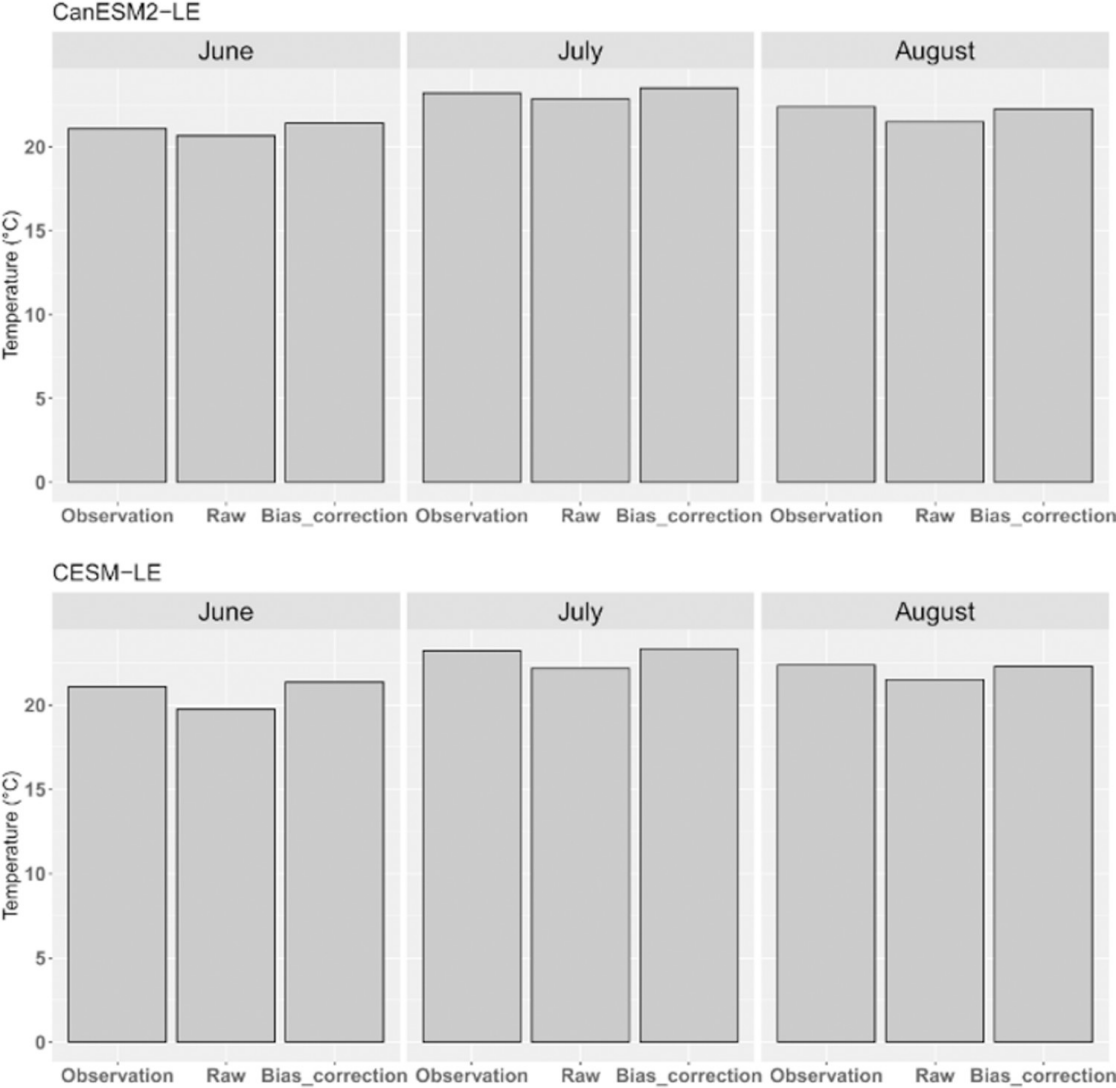
Regional mean temperatures in June, July and August during current climate (1.0°C warming relative to pre-industrial period) in observations, raw simulations and bias-corrected simulations in CanESM2-LE and CESM-LE. Noted that the multi-member mean was used in the evaluation.

The June-July-August (JJA) temperatures of the two sets before and after bias correction were compared with the observations, as shown in Fig 2. Compared to the observations, slight cold biases are observed in the raw simulation. The bias-corrected simulation reduces cold biases, especially in CESM-LE. For example, the observed August temperature is 22.4°C, the raw simulation of CESM-LE exhibit a cold bias with nearly 1.0°C compared to observation. The bias in bias-corrected simulation is decrease with the bias only 0.1°C.

**Fig 3:**
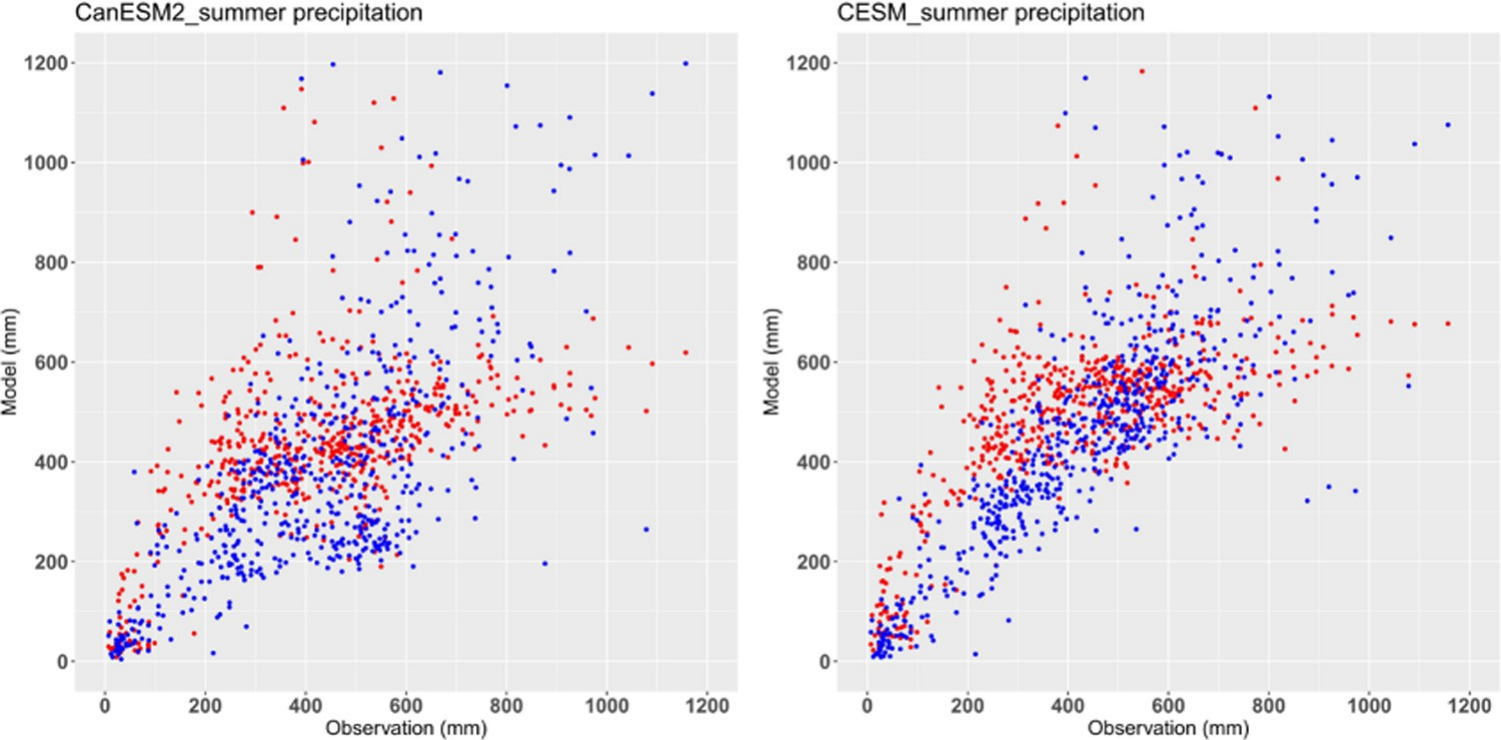
Simulated annual total precipitation in China during current climate in raw and bias-corrected simulation, where red represents the raw simulation and blue represents the bias-corrected simulation.

Fig 3 shows a comparison of the raw and bias-corrected total precipitation in JJA. The raw model underestimates heavy precipitation and overestimates light precipitation (red dots). The bias-corrected model simulates summer precipitation well. These results demonstrate that the bias correction of two ensembles can be used to conduct future projections of CDHEs over China.

#### 3.2. Future risk in compound dry and hot event

**Fig 4:**
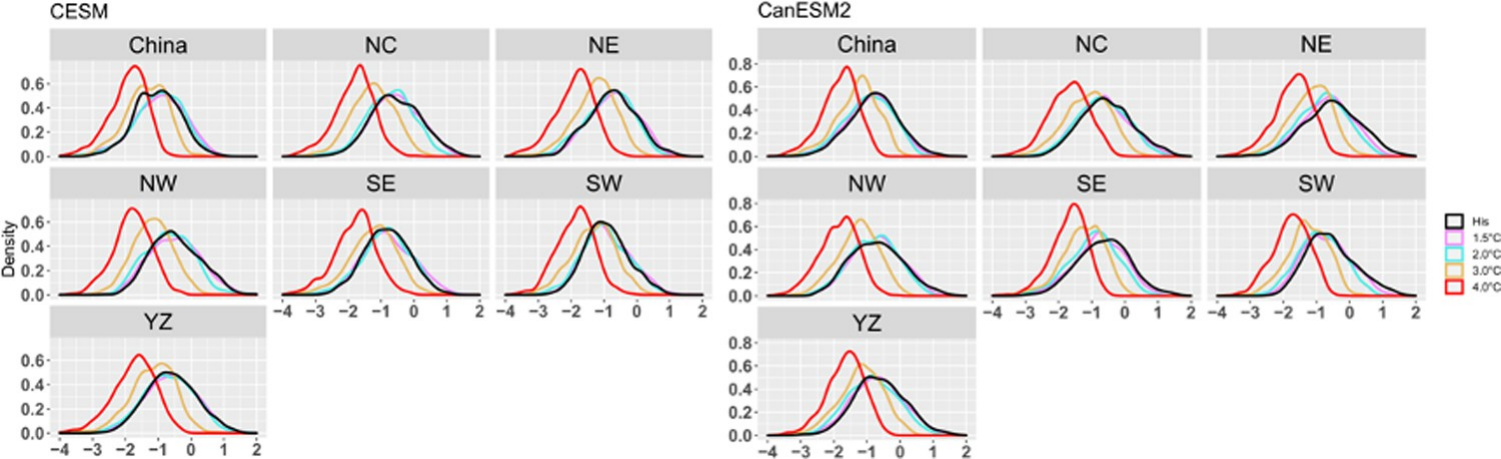
PDF of SDHI index for the China region and different subregions in a historical period and different future warming scenarios.

To study the changes in the frequency of CDHEs in China and its subregions under different warming backgrounds in the future, Fig 4 shows the probability density function (PDF) of the SDHI index under different warming scenarios of the current climate and future warming of 1.5°C, 2.0°C, 3.0°C and 4.0°C.

The SDHI shows little change under low warming climates (1.5°C and 2.0°C) in China and its six subregions. However, the PDF of the SDHI moves to the left sharply under a high warming climate (3°C, 4°C), which demonstrates that the probability of more extreme CDHEs will significantly increase across almost all of China. The results from the two large ensembles are consistent.

**Fig 5:**
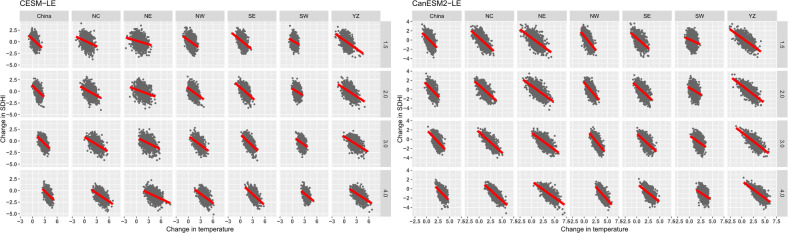
Scatter diagram of SDHI changes and temperature changes in China and its six subregions under different warming scenarios. The solid line represents the linear regression.

Fig 5 explores the relationship between two main driving factors, temperature and precipitation, and SDHI index changes at different warming levels in China and its subregions.

In general, there is a significant negative correlation between temperature change and SDHI change with an increase in global temperature. The magnitude of the increase in temperature in different subregions showed little difference, with a large increase in northern China (NC, NW and NC) and the YZ region. However, the changes in SDHI are very diverse between the two ensembles. For CESM-LE, three regions, NC NW and YZ, experience the largest decrease in SDHI, which means that the change in SDHI may be related to temperature. However, for CanESM2-LE, the changes in the SDHI show little diversity among different subregions. The difference response in the two ensembles may partly due to: 1) the key feedback processes determining the compound hot and dry event exhibit difference in the two ensembles. 2) The difference of horizontal resolution between the two ensembles.

For the relationship between SDHI and temperature, the consistent result of the two large ensembles is that the relationship between temperature and SDHI is worst in the SW region. However, a poor relationship in YZ is found in CESM-LE. In terms of the relationship between SDHI and temperature, scatter point distribution in the NE and NC regions is more dispersed compared to other regions, indicating weak correlation between them, whereas the negative correlation between SDHI and temperature is most significant in the NW and YZ regions, indicating a stronger response of SDHI to temperature change in these two regions. The influence of temperature on the SDHI is relatively weak in NE and NC but strong in NW and YZ, which demonstrates that temperature may play an important role in the decrease in the SDHI over the NW and YZ regions.

**Fig 6:**
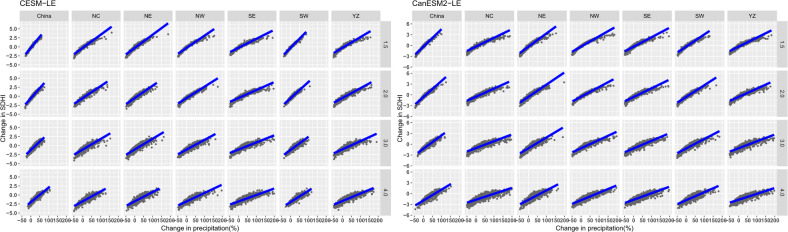
Same as Fig 5, but for the temperature changes and SDHI changes.

Similarly, the relationship between precipitation changes and SDHI change under different warming levels was also studied, and the results are shown in Fig 6.

Generally, the relation between SDHI changes and precipitation change shows little difference among the six subregions in both ensembles, which is more obvious in CanESM2-LE. A notable difference among subregions can be found in CESM-LE, with the strongest relationship in SW.

**Fig 7:**
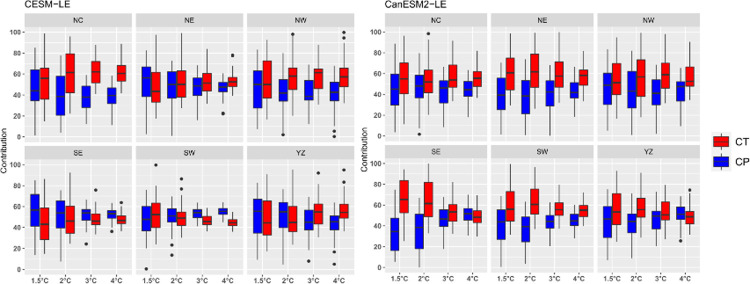
A boxplot of the contribution from temperature (CT) and precipitation (CP) to the changes in the SDHI in the six subregions. The upper and lower limits of the box indicate the 75th and 25th percentile values; the horizontal line in the box indicates the median; and the whiskers show the range of the ensemble.

The contributions of precipitation and temperature to the SDHI changes in the six subregions are displayed in Fig 7. In general, the contribution of temperature is greater than that of precipitation in almost all regions, especially under high warming scenarios. However, there are exceptions, such as SE and SW in CESM-LE, where the contribution of precipitation is always greater than that of temperature. The median contribution of precipitation in SE and SW are both nearly 55% under 4.0°C. The uncertainties in the contributions from temperature and precipitation decrease with global warming. Taking the SW in CESM-LE as an example, the contribution from temperature ranges from 30% to 60% under 1.5°C, this uncertainty range decreases from 45% to 50% under 4.0°C. The contribution of temperature in the NW and NC regions is significantly greater than that of precipitation in both large ensemble models. However, the characteristics of the remaining regions differed in each simulation. For example, in SE and SW in CanESM2-LE, temperature contributes significantly more than precipitation with above 60% for temperature, but there is little difference between the contributions of temperature and precipitation in CESM-LE, indicating that the contribution of precipitation is weaker than that of temperature.

In addition, the contributions of temperature are greater than those of precipitation over northern China. While the contributions of temperature and precipitation exhibit large differences over southern China. The northern region is relatively arid, the positive feedback effect of high-temperature drought is stronger, and the contribution of temperature is greater. However, in the humid south, this feedback effect is relatively weak [32].

**Fig 8:**
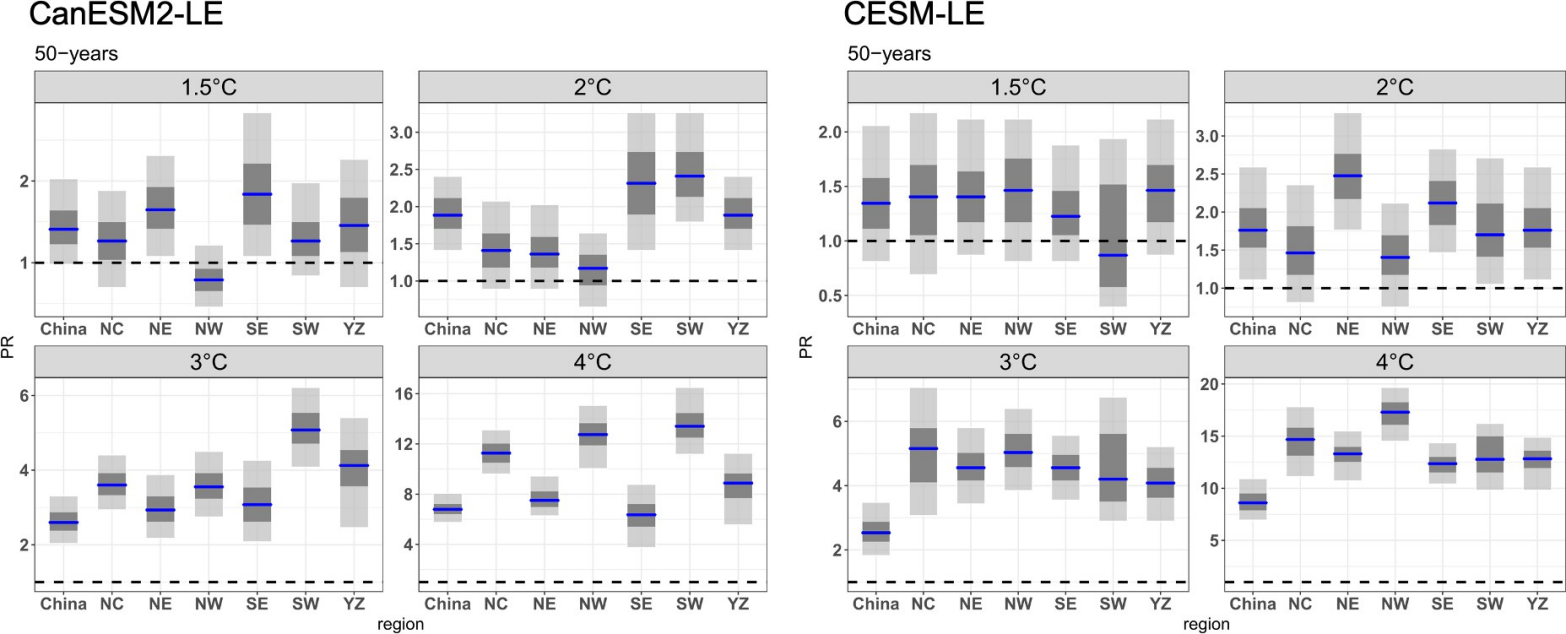
The risk ratio of CDHEs in 50 years under different warming scenarios. The blue line represents the risk ratio, and the grey indicates the confidence interval range obtained based on 1000 bootstrap samples. The dark grey demonstrates the 95% confidence level, and the light grey demonstrate the 90% confidence level. we have added in the revised manuscript.

The best estimation of the risk ratio and 90% and 95% confidence interval of CDHEs under different warming levels were determined in the two ensembles, and the results are shown in Fig 8. Compared with the current climate (1°C), the risk of CDHEs that occur once in 50 years under the 1.5°C warming scenario showed little increase in both ensembles, and the confidence range between 5% to 95% in almost all regions is less than 1.0, except NE and SE in CanESM2-LE. Under 2°C warming, the risk of CDHEs increased to a certain extent. However, the increase in SDHI over NC, NE, and NW in CanESM2-LE and NC and NW in CESM-LE was not obvious. Under a 3°C warmer climate, the risk of CDHEs increased significantly, with a PR larger than 1.0 in all subregions. The risk changes in CESM-LE were higher than those in CanESM2-LE, with the risk in all regions increasing by more than 4 times compared with the current climate in CESM-LE. The increase in risk in CanESM2-LE was relatively weak, and there were large regional differences, with the greatest increase in the SW where the risk increased by 5 times compared to the current climate. When the temperature rose by 4°C, the risk of CDHEs increased by more than 10 times in all the subregions in CESM-LE. NW received the greatest increase in the risk of CDHEs, with the probability of CDHEs increasing by more than 15 times over that of the current climate. The risk in CanESM2-LE increased slightly, with three regions, SW, NC and NW, experiencing the greatest increase in risk, with values larger than 10.

**Fig 9:**
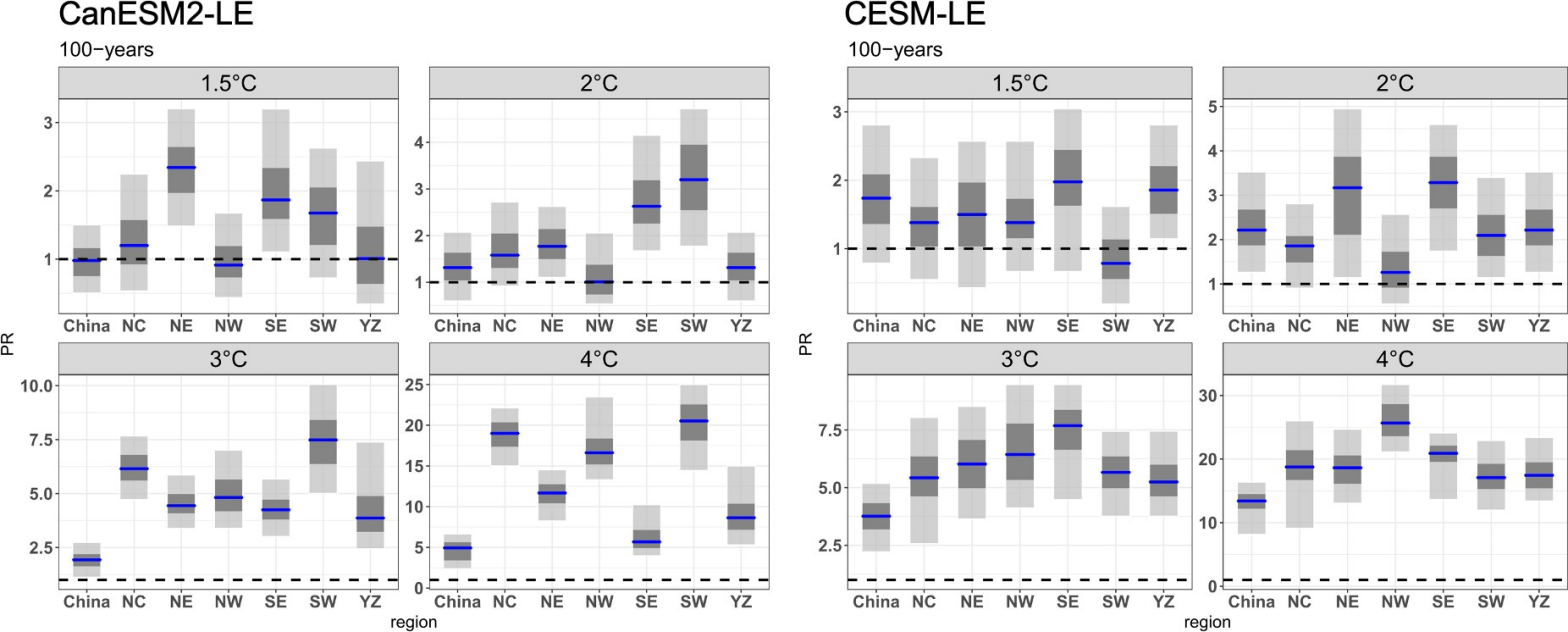
Same as Fig 8, but for the 100-years CDHEs.

The future risk changes of the 100-year CDHEs are displayed in Fig 9. Generally, the risk of events with 100-year return period shows a larger increase than that of event with 50-year return period. This result indicates that the more severe CDHEs will have a larger increase in the risk. The risk of events in NE and SE for CanESM2-LE and YZ for CESM-LE increases under a 1.5°C warmer climate. Under a 2.0°C warmer climate, SE and SW in CanESM2-LE and NE and SE in CESM-LE show the greatest magnitude of increase, with PRs greater than 3.0. With further warming, the risk in all subregions exhibits an increase in both ensembles with a PR larger than 5.0; that is, the events that occur once in 100 years will become more frequent, occurring once in 20 years in a 3.0°C warmer climate. Under a 4.0°C warmer climate, all subregions experience increasing risk in CDHEs, with the largest increase in NC, NW and SW for CanESM2-LE (nearly 20) and NW for CESM-LE (nearly 25). This means that the extreme CDHEs expected once every 100 years in the current period over NW are expected to occur approximately every 5 and 4 years in CanESM2-LE and CESM-LE, respectively.

## 4. Conclusion and discussion

In this paper, two sets of large ensemble simulations, CanESM2-LE and CESM-LE, were used to estimate the occurrence risk of extreme compound hot and dry events under different warming backgrounds in China. A quantile mapping-based method was first used to reduce the biases in simulating summer precipitation and temperature. The index SDHI was constructed based on a copula function to represent the severity of compound hot and dry events. The contributions of temperature and precipitation to the SDHI were also investigated. The conclusions were as follows:

1. The frequency of extreme CDHEs in China exhibits little increase under a low warming climate. However, the probability of extreme CDHEs shows quite a large increase in magnitude under a high warming climate.
2. The more extreme events will have a greater risk of occurrence in the future, but the uncertainty is also larger. Events that would occur once every 100 years in the current climate in CESM-LE (CanESM2-LE) will be 1.3/2.3 (1.5/2.0) times more likely to occur in a 1.5°C/2.0°C warmer climate, respectively. Northwestern China will experience the greatest increase in the risk of extreme CDHEs. Extreme CDHEs expected once every 100 years in the current period over NW are expected to occur approximately every 5 and 4 years under a 4.0°C warmer world in CanESM2-LE and CESM-LE, respectively.
3. The future increase in extreme CDHEs mainly results from the increase in temperature. The contribution of temperature to the CDHEs in northern China is significantly greater than that of precipitation in both large ensemble simulations.

There are three main uncertainties for the future projection: 1) internal variability uncertainty; 2) model structure uncertainty and 3) scenario uncertainty. We used two models to give the projection of compound dry and hot event in this article. The two models exhibit quite a large diversity in changes of CDHEs. Thus, more modes with large ensembles are needed to study the projection of CDHEs stem from model uncertainty. In addition, dynamical downscaling providing more accuracy of key processing determining the compound event is expected to give accurate projection of CDHEs [33].

These two large ensembles give only the projection under RCP8.5 scenario closing to the current emission scenario. Thus, it is important to study the projection uncertainty stems from different emission scenario.
